# Tamoxifen-Induced Apoptosis of MCF-7 Cells via GPR30/PI3K/MAPKs Interactions: Verification by ODE Modeling and RNA Sequencing

**DOI:** 10.3389/fphys.2018.00907

**Published:** 2018-07-11

**Authors:** Milad Rouhimoghadam, Shahrokh Safarian, Jason S. Carroll, Nader Sheibani, Gholamreza Bidkhori

**Affiliations:** ^1^Department of Cell and Molecular Biology, School of Biology, College of Science, University of Tehran, Tehran, Iran; ^2^Cancer Research UK Cambridge Institute, University of Cambridge, Cambridge, United Kingdom; ^3^Department of Ophthalmology and Visual Sciences, Biomedical Engineering, and Cell and Regenerative Biology, University of Wisconsin School of Medicine and Public Health, Madison, WI, United States; ^4^Science for Life Laboratory, KTH Royal Institute of Technology, Stockholm, Sweden

**Keywords:** tamoxifen, GPR30, ODE modeling, RNA sequencing, apoptosis, MCF-7

## Abstract

Tamoxifen (Nolvadex) is one of the most widely used and effective therapeutic agent for breast cancer. It benefits nearly 75% of patients with estrogen receptor (ER)-positive breast cancer that receive this drug. Its effectiveness is mainly attributed to its capacity to function as an ER antagonist, blocking estrogen binding sites on the receptor, and inhibiting the proliferative action of the receptor-hormone complex. Although, tamoxifen can induce apoptosis in breast cancer cells via upregulation of pro-apoptotic factors, it can also promote uterine hyperplasia in some women. Thus, tamoxifen as a multi-functional drug could have different effects on cells based on the utilization of effective concentrations or availability of specific co-factors. Evidence that tamoxifen functions as a GPR30 (G-Protein Coupled Receptor 30) agonist activating adenylyl cyclase and EGFR (Epidermal Growth Factor Receptor) intracellular signaling networks, provides yet another means of explaining the multi-functionality of tamoxifen. Here ordinary differential equation (ODE) modeling, RNA sequencing and real time qPCR analysis were utilized to establish the necessary data for gene network mapping of tamoxifen-stimulated MCF-7 cells, which express the endogenous ER and GPR30. The gene set enrichment analysis and pathway analysis approaches were used to categorize transcriptionally upregulated genes in biological processes. Of the 2,713 genes that were significantly upregulated following a 48 h incubation with 250 μM tamoxifen, most were categorized as either growth-related or pro-apoptotic intermediates that fit into the Tp53 and/or MAPK signaling pathways. Collectively, our results display that the effects of tamoxifen on the breast cancer MCF-7 cell line are mediated by the activation of important signaling pathways including Tp53 and MAPKs to induce apoptosis.

## Introduction

Breast cancer is the second leading cause of cancer death in the United States in women after lung cancer (Holt, 2010). Clinically, breast cancer is categorized as ER-alpha positive and ER-negative (Haldosen et al., 2014), which dictates the treatment options. The GPR30 receptors are well-known members of the large family of GPCR (G protein–coupled receptors) receptors whose sequences are located in the 7P22.3 of human genome (Albanito et al., 2008). These receptors are structurally different from estrogen receptors (ER) and are expressed extensively within early mammary tumors and in breast cancer cell lines (Prossnitz et al., 2008; Prossnitz and Barton, 2009). Activation of the GPR30 signaling cascade mediates the non-genomic route of estrogen hormones and stimulates the PI3K/Akt and MAPK (Mitogen-Activated Protein Kinase) signaling intermediates. Thus, this pathway controls different cellular processes that can culminate in cell proliferation, migration, differentiation, and apoptosis (Filardo et al., 2000; Ge et al., 2012; Luo et al., 2012). Besides interacting with estrogenic hormones, the receptor GPR30 also has a tendency to bind a number of anti-estrogenic compounds such as tamoxifen and ICI182780 (Thomas et al., 2005).

Tamoxifen is a non-steroidal compound that is used extensively in patients with ER+ breast cancer. In addition to its beneficial effects in reducing metastasis, tamoxifen can also lower the risk of death from breast cancer (Jordan, 2006; Jordan and O’Malley, 2007). Studies have shown that at low concentrations, tamoxifen represses the cell cycling process, while at higher concentration it induces cell apoptosis (Osborne et al., 1983; Perry et al., 1995; Chen et al., 1996; Otto et al., 1996).

Activation of the GPR30 signaling pathway can stimulate the PI3K and MAPK signaling pathways. In this way, after binding of the ligand to the GPR30 receptor, the subunit Gα separates from the subunit Gβγ, and can activate the adenylyl cyclase and phospholipase C. The Gβγ subunit itself activates the Src-like tyrosine kinase protein, which induces the phosphorylation of Shc protein and activates the matrix-metalloproteases (MMPs). The activated MMPs trigger the transactivation of EGFR receptor by cleaving the HB-EGF (Heparin Binding EGF) in the membrane, and ultimately releasing EGF. This leads to temporary activation of ERKs, which subsides after the signal diminishes (Luttrell et al., 1999; Filardo et al., 2000; Filardo, 2002).

The Ras/Raf/ERKs signaling pathway is one of the most commonly deregulated pathways in a variety of tumors. Many studies have shown that, depending on the duration and its subcellular localization, ERK activity controls various cellular responses such as proliferation, migration, differentiation, and apoptosis (Murphy and Blenis, 2006). Apoptosis is triggered by certain conditions including hyperactivity of ERKs. Interestingly, active ERK can be located within the mitochondrial membrane and facilitate the cytochrome c entrance into the cytoplasm by disrupting the mitochondrial membrane (Leeman et al., 2006). Also, the MEK/ERKs pathway activity can upregulate the pro-apoptotic members of Bcl-2 family such as Bax, PUMA, and Bak as well as down regulate the anti-apoptotic members of the family such as Bcl-2 and Bcl-XL (Li et al., 2007; Liu et al., 2008; Panaretakis et al., 2008). In addition, ERK-dependent over-activation of Tp53 protein leads to phosphorylation of serine 15 residue in Tp53, and inhibits the Mdm2 interaction with Tp53. This results in accumulation of Tp53. Studies have shown that ERK can phosphorylate threonine 55 residue of Tp53, increasing its binding to DNA and down regulating Bcl-2 gene expression (Persons et al., 2000; She et al., 2000). Overall, these data suggest that sustained ERK activity in some cellular regions is not tolerated and leads to different forms of cell death (Cagnol and Chambard, 2010).

In the current study, we aimed to simulate the GPR30/PI3K/MAPK/STAT signaling pathway in normal and cancer cells by the use of ordinary differential equation (ODE) modeling. In addition, we planned to show that MAPK activation pattern is different between normal and cancer models. We also assessed the activity of ERK in tamoxifen-treated and untreated cells using RNA-sequencing and real time RT-PCR (qPCR) methods. The expression level of the known apoptosis signaling intermediates were examined in the MCF-7 cells incubated with tamoxifen to ascertain the relationship between tamoxifen-induced MAPK signaling pathway and induction of apoptosis in these cells.

## Materials and Methods

### Materials

Roswell Park Memorial Institute Medium 1640 (RPMI 1640), tamoxifen citrate salt, 3-[4,5-dimethylthiazol-2-yl]-2,5-diphenyl tetrazolium bromide (MTT) and Dimethyl sulfoxide were all obtained from Sigma (United States). Fetal bovine serum (FBS) was from Invitrogen (United States). Annexin-V-FLUOS Staining Kit, TriPure Isolation Reagent, Streptomycin and penicillin, Propidium Iodide (PI) kit, 4,6-diamidino-2-phenylindole (DAPI) kit were provided from Roche (Germany). RealQ Plus Master Mix Green was provided from Ampliqon (Denmark). cDNA generation was carried out with RevertAid First Strand cDNA Synthesis kit by Thermo Scientific (United States).

### Computational Modeling of Signaling Pathway

Modeling is one of the branches of system biology, which is able to simulate a simple or a series of related reactions within an organism based on mathematical equations. Using this approach, the biological events could easily transform into quantitative data and subsequent analysis. ODE is one of the most widely used methods for modeling a bionetwork. In general, the ODE relationship represents how the concentration of a substrate changes over time (Kitano, 2002; Kirschner, 2005; Bidkhori et al., 2012).

One of the most exciting developments in signal transduction research is the use of mathematical models and computer simulation in the field of pharmacology and drug discovery. Signal transduction networks are largely composed of proteins which can interact, move to specific cellular locations, or be modified or degraded. An advantage of ODE models is that they describe the rate of change of continuous variables which used for modeling dynamical systems in several areas (Eungdamrong and Iyengar, 2004; Chen et al., 2010; Hughey et al., 2010). In this work, mass-action laws are used to model the dynamics of systems of chemical reactions, that is, reaction networks.

For example, a substrate (S) is converted to the product (P) with a constant rate (k1) that can be calculated by the Mass Action Equation:

$$S\overset{k1}{\to }P\,v=k1\left[S\right]$$

This relationship shows a first-order reaction and indicates the direct linkage between the reaction velocity (V) and the first potential substrate concentration. In fact, the higher concentration of substrate boosts the reaction rate and substrate consumption resulting in faster production of the product (P) (Chaudhury and Igoshin, 2010; Chen et al., 2010). According to the above equations, the differential relations for substrate and product will be as follows:

$$\frac{d\left[S\right]}{dt}=-k1[S]or\,\frac{d\left[P\right]}{dt}=k1\left[S\right]$$

To model the above reaction, it is important to know the initial concentration of the substrates and the products. Plus, quadratic reactions may also exist in these relationships. Thus, in these cases the reactions have a direct relationship with the concentration of two reactants. For modeling and simulating above reaction, it is necessary to know S and P initial values. The reaction above is called a first-order reaction. In such reactions, the rate is relational to the value of a single reactant. On the other hand, a second-order reaction is relational to the square of the value of an individual reactant or two reactants:

$$S+R\overset{k2}{\to }P\,and\,v=k2[S]\left[R\right]$$

k1 and k2 are defined in S^-1^ and μM^-1^S^-1^ dimensions respectively.

Our proposed model for the GPR30/EGFR/PI3K/STAT signaling pathway is based on an ODE modeling, which includes 128 species, 143 reactions, 213 parameters and 1 rule. In **Supplementary Table S1**, reactions are shown for this signaling pathway. To simulate the ODE15s routine, the MATLAB 7.9.0 was used to determine the differential equations. The systems biology markup language (SBML) of our models is provided in **Supplementary File S1** (untreated model) and **Supplementary File S2** (tamoxifen-treated model). SBML is a computer-readable XML format for representing models of biochemical reaction which allows models of arbitrary complexity to be represented.

The following relationship is an example of the assessment of simulated reactions using the ODE method in this paper:

Akt + PIP3 ​⇄kr1k1​​​​ Aktm

$$\text{v = k1}[\text{Akt}][\text{PIP3}]-{\text{k}}_{\text{r}}1\left[\text{aktm}\right]$$

Where k1 is the rate constant for the forward direction and kr1 is for the reverse one.

### Cell Culture and Treatment

Human breast cancer cell line, MCF7 cells (ATCC^®^ HTB-22^TM^), were purchased from Iranian Biological Resource Center (Tehran, Iran). Cells were cultured in RPMI-1640 medium (Sigma, United States) containing 100 μg/ml penicillin, 100 μg/ml streptomycin, 2 mM glutamine, 10% heat-inactivated FBS (Invitrogen, United States), and incubated at 37°C in a humidified 5% CO2 incubator. Heat-inactivation (heating to 56°C for 30 min) is done to inactivate complement, a group of proteins present in sera that are part of the immune response. This is sometimes important for cells that will be used to prepare or assay viruses, or cells that are used in cytotoxicity assays or other systems where complement may have an unwanted influence. In our work, heat-inactivated serum was used in the experimental medium in order to inactivate complement and diminish the content of estrogens. According to MTT assay, the LC50 of tamoxifen after 48 h was determined as 250 μM. Tamoxifen was dissolved in DMSO and then added to culture medium to the final desired concentration based on the determined LC50 and filtered. Cells (at 80% confluency) were incubated with freshly prepared drugs for 48h in a humidified incubator before being trypsinized and washed with phosphate-buffered saline three times and stored at -70°C.

### MTT Assay (Cytotoxicity/Viability Assay)

Cell viability was assessed after tamoxifen treatment using the 3-(4,5-dimethylthiazol-2-yl)-2,5-diphenyltetrazolium bromide (MTT). Twenty microliter of 0.5-mg/mL concentration of MTT stock (diluted in PBS, pH 7.2) was added to the culture medium and cells were incubated at 37°C with 5% CO2 for 3 h. Next, the cells were incubated with 200 μl of dimethyl sulfoxide (DMSO; Sigma) for an additional 5 min, and their viability was measured using a microplate reader (Rayto-China) at 570 nm. The MTT assays were performed at least three times for each concentration of drug and the percentage of surviving cells relative to control (untreated sample) was determined.

### Quantification of Cell Cycle Distributions and Apoptosis by Flow Cytometry

Flow cytometry was used for the evaluation of apoptosis in treated cells. Annexin V-FITC and propodium iodide (PI) staining was performed using Annexin-V-FLUOS and PI staining kit, followed by flow cytometry using a Becton Dickinson instrument (United Kingdom). Cells were incubated with 250 μM of Tamoxifen for 48 h. Cells (10^6^) were washed in PBS and suspended in 100 μl Annexin/PI buffer (20 μl of each Annexin and PI buffer in 1 ml of incubation buffer) for 10–15 min at 25°C. After dilution in 500 μl of incubation buffer, fluorescence was measured at excitation and emission wavelengths of 518 and 617 nm, respectively. Cell cycle distribution was determined using the DAPI staining kit. Cells (5 × 10^5^) were incubated with 1 ml of fluorochrome solution (10 μg/ml, DAPI; and 6% triton X-100 in PBS) in the dark for 30 min at 4°C. Fluorescence was recorded at excitation and emission wavelengths of 359 and 461 nm, respectively, using the Becton Dickinson flow cytometer. Data was analyzed on 2-dimensional curves (number of cells against area under the peak), using FlowJo software.

### RNA Extraction

Total RNA was extracted from the cells using TriPure Isolation Reagent (Roche Diagnostics GmbH, Germany) according to the kit’s protocol. To avoid genomic DNA contamination, the RNA samples were treated with DNase for one hour. The RNA concentration was measured using a Nano drop ND-1000 spectrophotometer (Nano drop Technologies), and the purity was assayed by measuring the A260/A280 ratio, which was expected to be 1.8–2.0.

### cDNA Library Construction and RNA Sequencing

Following RNA isolation, creation of an RNA-seq library is the next step in transcriptome sequencing. The preparation of intended cDNA libraries and paired-end sequencing were performed by the Beijing Genome Institute (BGI), China. In total, four cDNA paired-end libraries were generated for transcriptome sequencing on Illumina HiSeqTM 2000 platform.

Briefly, rRNA depletion methods were used for library construction. Based on the protocol, after rRNA depletion all other RNAs with or without the polyA sequence were subjected to adaptor ligation. The adapter-ligated templates were purified by the Agencourt AMPure SPRI beads and fragments with insert size about 200 bp were excised and used to synthesize the first strand cDNA. The second-strand cDNA was synthesized using dNTPs, RNaseH and DNA polymerase I. Suitable fragments were amplified by ligation-mediated polymerase chain reaction (LM-PCR), purified, and hybridized to the SureSelect Biotinylated RNA Library (BAITS) for enrichment. Hybridized fragments were bound to the streptavidin beads whereas non-hybridized fragments were washed out after 24 h. Captured LM-PCR products were subjected to Agilent 2100 Bio analyzer to estimate the magnitude of enrichment. Each captured library was then loaded on HiSeq 2000 platform, and subjected to high-throughput sequencing for each captured library independently to ensure that each sample met the desired average fold-coverage. Raw image files were processed by Illumina base calling Software 1.7 for base calling with default parameters and the sequences of each individual were generated as 90 bp paired-end reads. The obtained raw data were subjected to the GO analysis (version 6.8, 2016) and KEGG mapping for pathway analysis of the screened DEGs. The data discussed in this publication have been deposited in the Gene Expression Omnibus (GEO) database, https://www.ncbi.nlm.nih.gov/geo and are accessible through GEO Series accession number GSE115737.

### Reverse Transcription and Quantitative Real-Time PCR Analysis (RT-qPCR)

RNA samples (2 μg) were subjected to cDNA synthesis by the RevertAid First Strand cDNA Synthesis kit. The cDNA samples were amplified using the RealQ Plus Master Mix Green Kit with QIAGEN’s real-time PCR cycler, the Rotor-Gene Q with an initial denaturation at 95°C for 15 min followed by 45 cycles each 95°C for 15 s, 62°C for 10 s, and 72°C for 20 s. Relative expression abundance of targets were determined by Relative expression software tool (REST) for group-wise comparison and statistical analysis of relative expression results in real-time PCR.

### Statistical Analysis

Distribution and quality of the RNA-seq data sets were assessed using various statistical methods. Using the formula: Phred Quality (Q) score = -10log2E, the error rate of data sets was calculated (E stands for error rate). Guanine-cytosine (GC) pairs distribution were determined and the homogeneity of the sequences was confirmed. The unwilling raw reads were excluded from subsequent analysis including adaptor sequences, reads with low quality, raw reads contain more than 20% N bases or those with more than 50% N bases that achieved the Q-score above the 20.

The Cufflinks software (version 2.2.1, 2014) was applied to evaluate the expression level of intended genes. The expression score was calculated by the formula: RPKM = total exon reads/mapped reads in millions × exon length in kb, which RPKM stands for reads per kilobase of transcript per million mapped reads. Also, using the DESeq software (version 1.12.1, 2016) the difference between experimental groups was analyzed.

Comparisons between the groups were made by a one-way analysis of variance (ANOVA) followed by an appropriate *post hoc* test to analyze the difference. All data are represented as the mean ± SD (Standard deviation). The *P*-values correction was done by the Benjamini–Hochberg multiple testing. Statistical significance was achieved when *P* and *q* values were <0.05. All statistical analyses were performed with IBM SPSS Statistics software version 22 (IBM, United States).

## Results

### Construction of a Model for ERK Activation Through GPR30 Axis

**FIGURE 1:**
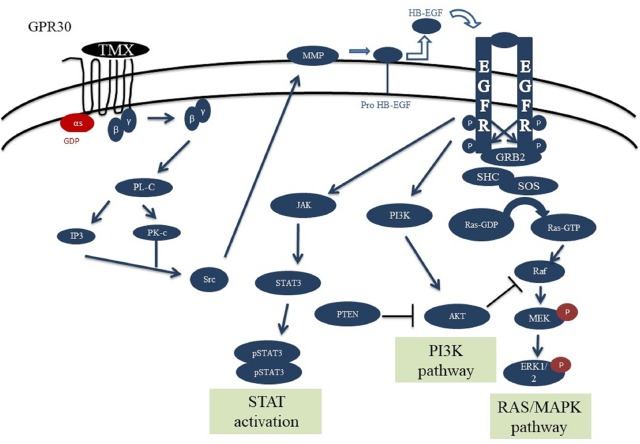
Schematic overview of the GPR30/EGFR/PI3K/STAT signaling axis. This network consists of the interaction between GPR30/PI3K/MAPK/STAT pathways. Initial stimulation by tamoxifen causes activation of GPR30 receptors and activation of PLC by releasing the Gβγ subunit which can trigger ERK activation. Also, src can activate MMPs which can convert HB-EGF to EGF. EGF can bind and activate EGFR, causing receptor dimerization and cross-phosphorylation of tyrosine residues in the intracellular domains. The activated EGFR axis can phosphorylate ERK and through that regulates various cell processes. PI3K and JAK can be recruited to cell membrane by interaction with EGFR phosphotyrosine docking sites. PI3K subsequently causes AKT activation and regulates cell growth and survival. Activation of STAT dimers by JAK play a key role in controlling cell growth and survival. Since JAK-STAT signaling can allow the transcription of genes involved in cell division, one potential effect of excessive JAK-STAT signaling is cancer formation.

The designed signaling network for normal cells is modeled based on the experimental evidences and previous models of the EGFR, PI3K, STAT and GPCR signaling pathways (Schoeberl et al., 2002; Yamada et al., 2003, 2004; Sasagawa et al., 2005; Heitzler et al., 2012). This network consists of four main pathways (**Figure 1**), which play important roles in cell proliferation, differentiation, and apoptosis. These pathways are activated through two ligands alongside the two axes: 1- through the EGF binding to EGFR, and 2- via tamoxifen binding to GPR30 (**Supplementary Table S1**).

After binding of EGF to EGFR, the receptor is formed into the hetero- or homo-dimeric state, which leads to auto phosphorylation of tyrosine resides including pY992, pY1068 and pY1173 at the C-terminal region (Walton et al., 1990). Proteins such as Grb2, Shc and STAT can bind to the phosphorylated tyrosine residues. Following C-terminal phosphorylation of EGFR, the Shc protein is bound and provokes Grb2 and SOS accumulation. Grb2 can interact with the receptor alone and invoke SOS recruitment. SOS then converts Ras-GDP into Ras-GTP, which is the active form of Ras. The Ras-GTP binds to the serine/threonine kinase Raf and activates it. Subsequently, Raf stimulates MEK (MAP kinase kinase) via phosphorylation. The activated MEK phosphorylates ERK and through that regulates various cell processes such as cell growth or death (Marais et al., 1995; Wiley et al., 2003; Steelman et al., 2011).

The PI3K has a regulatory subunit (85 kDa) along with a catalytic subunit (110 kDa). After phosphorylation and activation of EGFR, the regulatory subunit connects to the phosphorylated tyrosine residue, then catalytic subunit is bound to the regulatory unit and induces the membrane Phosphatidylinositol 4,5-bisphosphate [PI(4,5)P2] conversion into the Phosphatidylinositol (3,4,5)-trisphosphate [PI(3,4,5)P3]. Next, the PI3,4,5P3 binds and stimulates the PDK1 protein to phosphorylate AKT. AKT regulates cell growth and survival in a direct or an indirect manner. Negative adjustment of PI3K as well as deactivation of AKT are accomplished by the protein phosphatase PTEN, which converts PI(3,4,5)P3 into PI(4,5)P2 and subsequently causes AKT suppression (Franke et al., 1997; Silva et al., 2008).

Signal transducer and activator of transcription are specific transcription factors that act as downstream effectors in the cytokine and growth factor receptors signaling pathways. STAT proteins can directly bind EGFR via their SH2 domains and form active phosphorylated dimers. STAT dimers play a key role in controlling cell growth and survival by regulation of the target genes (Leeman et al., 2006; Mertens and Darnell, 2007).

The other axis of our model consisted of the GPR30 receptor signaling pathway. The GPR30 is activated by binding of the tamoxifen and boosts the activation of PLC by releasing the Gβγ subunit. PLC stimulates the IP3 and DAG production from the membrane PI(4,5)P2. Both IP3 and DAG activate PKC, which can trigger ERK activation. The suppression of GPCR (GPR30) is mediated by phosphorylation with the GRK. The phosphorylation of GPR30 by GRK causes binding of β-arrestin protein to the receptor and internalization of the formed complex. This complex is separated into its constituent elements in the endosomes environment with low pH, and the receptor subunit returns to the cell membrane after dephosphorylation. Studies show that GRK families have different variants, as some of them cause the formation of a complex and evoke the β-arrestin 2 factor, which mediates the phosphorylation of ERKs independent of the GPR30 receptors (Heitzler et al., 2012).

### ODE Modeling and Comparison of ERK Activity in the Experimental Groups

**FIGURE 2:**
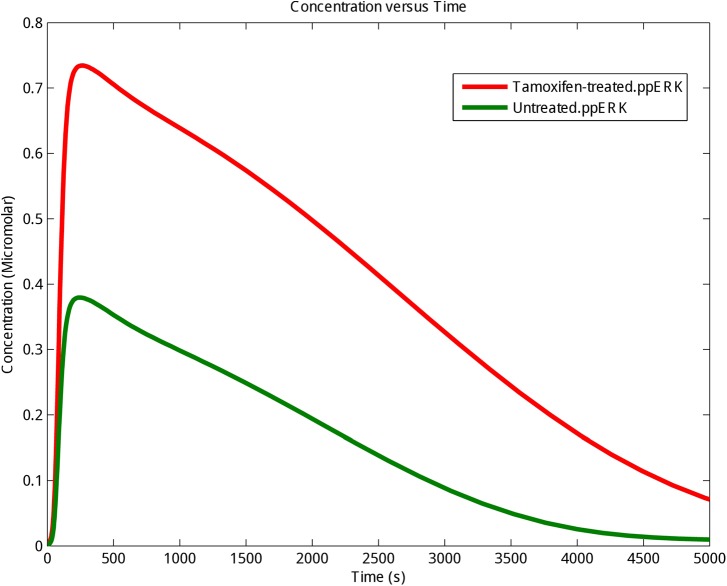
The kinetics of phosphorylation and activation of ERK protein. The activating phosphorylation of ERK (ppERK) was enhanced under tamoxifen stimulation. Control (Untreated cells): Green graph; Tamoxifen-treated cells: Red graph.

ERK is a known pro-survival factor that can also have pro-apoptotic role in cancers. Extensive ERK activity has been observed in 84% of prostate cancer patients, 91% of head and neck cancer cases, 67% of gastric cancers, and 72% of the breast cancer population. However, others have shown that sustained ERK activity can lead to apoptosis (Gioeli et al., 1999; Gee et al., 2001; Murphy and Blenis, 2006). Also, P-ERKs have been reported in 48% of renal and 58% of hepatocellular carcinoma cancerous samples, respectively (Oka et al., 1995; Ito et al., 1998). Our computational modeling showed that stimulation of GPR30 with tamoxifen, changed ERK activity in MCF-7 cells (**Supplementary Table S1**). As already demonstrated in **Figure 2**, the concentration and stability of the phosphorylated ERKs were significantly greater in the tamoxifen-treated group than the control group. Moreover, our simulation results demonstrate that enhanced activity of GPR30 signaling pathway has a considerable effect on MAPK pathway. Next, we took advantage of RNA sequencing and qRT-PCR methods in order to describe that MAPK pathway may play an important role in inducing apoptosis in MCF-7 cells.

**FIGURE 3:**
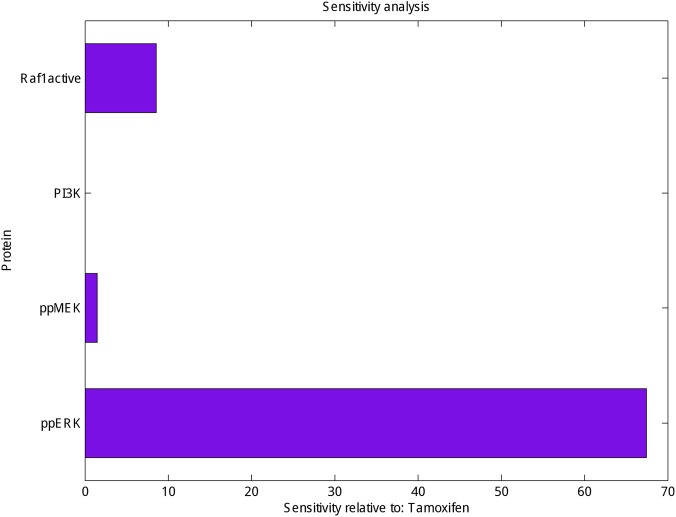
Sensitivity analysis of Raf1 active, PI3K, ppMEK and ppERK. Calculation the sensitivity of Raf1 active, PI3K, ppMEK and ppERK (*X*-axis) with respect to tamoxifen (*Y*-axis) which represented the highest sensitivity for ppERK.

Finally, sensitivity analysis as a commonly used approach that studies the response of system variables to changes in parameter values, and can therefore be used to identify the key reactions and species was carried out for PI3K, Raf1 active, ppMEK and ppERK in the presence of tamoxifen. As shown in **Figure 3** ppERK is more sensitive than PI3K, Raf1 active and ppMEK in simulated model.

### Incubation of MCF-7 Cells With Tamoxifen Results in a Dose-Dependent Decrease in Viability

**FIGURE 4:**
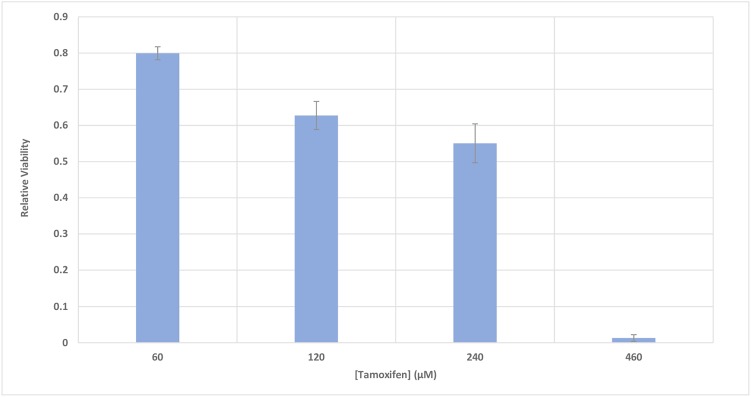
The relative viability of MCF-7 cells incubated with various concentrations of tamoxifen. The viability of the treated cells is shown to be different from the control in the presence of different concentrations of tamoxifen (60–460 μM). The incubation time of the treatments was 48 h. It can be seen that 50% decrease in the viability of the cells can be achieved approximately with 250 μM of tamoxifen and this is utilized in conjunction with all treatments here for induction of apoptosis in the cells.

The metabolic activity of MCF-7 cells was investigated based on the activity of enzyme succinate dehydrogenase. To accomplish this, the MTT method was used to determine the relative viability of the cells 48 h after incubation with different concentrations of tamoxifen (60–460 μM), with a goal of inducing cell apoptosis. **Figure 4** shows a 50% decrease in the viability of the cells incubated with 250 μM of tamoxifen.

### Induction of Apoptosis in MCF-7 Cells Incubated With Tamoxifen

**FIGURE 5:**
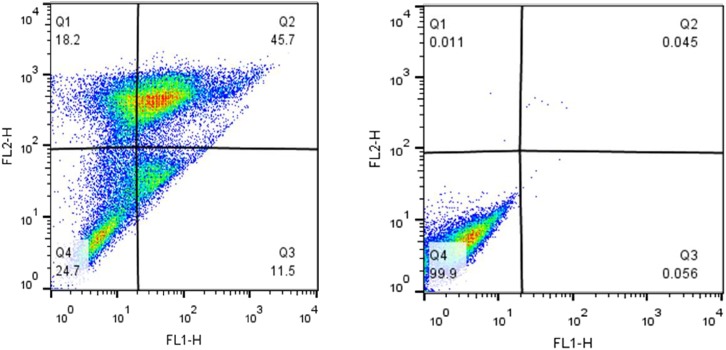
The impact of tamoxifen on apoptosis of MCF-7 cancer cells. Quadrants were drawn based on FSC/SSC plot in the untreated cells. Percent apoptosis was determined using two dimensional plot of PI (PI, *Y*-axis) and Annexin V-FITC (*X*-axis). **(Left)** Treated cells with a single dose of tamoxifen (250 μM), and **(Right)** the untreated control. Different cellular distributions are shown in the quadrants, Q1, necrotic cells; Q2, late apoptotic cells; Q3, early apoptotic cells; and Q4, normal cells.

Apoptosis induction was investigated using Annexin V and PI staining. The stained cells were classified into 4 groups, including Q1: Necrotic cells (Anx^-^, PI+), Q2: Late apoptotic cells (Anx+, PI+), Q3: Early apoptotic cells (Anx+, PI^-^) and Q4: Normal cells (Anx^-^, PI^-^). Incubation of MCF-7 cells with 250 μM tamoxifen resulted in their accumulation mainly in the Q2 region. The late stage in apoptotic cells showed accumulation of PI in the nucleus, and exposure of the phosphatidylserines in the outer leaflet of plasma membrane. This indicates that after 48 h, the number of the cells in late apoptotic phase had a significant increase from 0.045% in the control to 45.7% in cells incubated with tamoxifen. Therefore, induction of apoptosis was confirmed in MCF-7 cells incubated with tamoxifen (**Figure 5**).

### Cell Cycle Assessments

**FIGURE 6:**
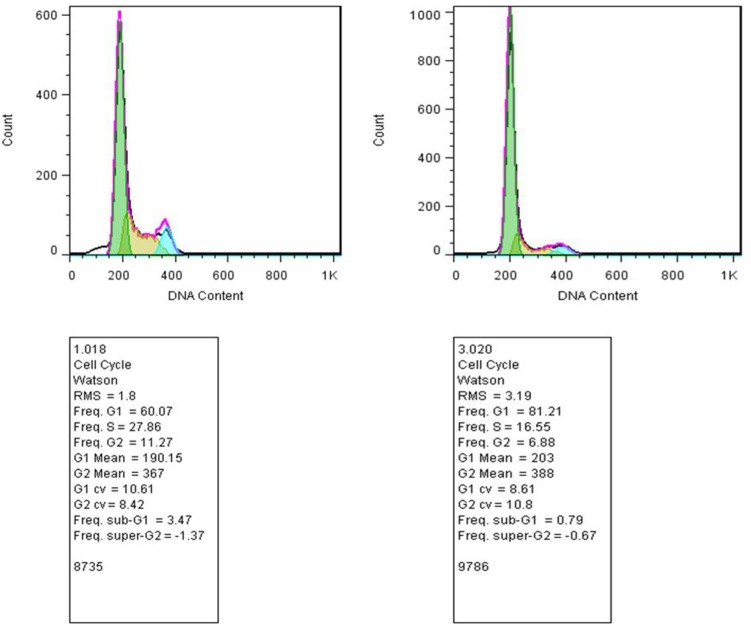
Study of the cell distribution in different cell cycle stages. **(Left)** MCF-7 cells incubated with tamoxifen, and **(Right)** control cells. The bell-shaped curves from left to right correspond to the G1, S, G2/M phases of the cell cycle in the control and cells incubated with tamoxifen. Incubation of cells with tamoxifen did not significantly shift the distribution of the cells in different phases of the cell cycle compared with control. As in the treated group, there were minor increases, about 10 and 6%, in the S and G2 phases of the cell cycle, respectively.

Our studies did not show any significant changes in the cellular distributions during different phases of the cell cycle (G1, S, G2) compared with control. Thus, emphasizing that the 50% decrease in cell viability after 48 h of incubation with tamoxifen did not originate from cell cycle arrest (**Figure 6**). Incubation of the cells with tamoxifen resulted in a 10% increase in the S phase and 6% in the G2 phase populations. These results suggest that the tamoxifen mediated reduced cell viability is not mediated by cell cycle arrest.

### Quality Evaluation of RNA Sequencing Data and Read Mapping

*Q*-score analysis showed that the quality of RNA-seq data had the required standards and can be used for further analysis. In addition, the N-base content was at the acceptable range of sequence data and well filtrated for further analysis. For RNA sequencing contents, we prepared two cDNA libraries (each consisted of two biological replicates) of tamoxifen-treated MCF-7 cells and control group. The libraries were sequenced using the Illumina HiSeq platform technology (BGI, China) and in total, 130,791,283 raw reads were obtained. After removing adapters and low-quality readings, we obtained 122,193,815 usable reads (**Table 1**). Next, the refined data were aligned with the human genome.

**Table 1:** Evaluation of valid reads mapped to the reference genome of each sample.

	Sample name	All reads	Concordantly 1	Concordantly more 1	Discordantly 1	Discordantly more 1	Overall alignment rate
1	Untreated-1	31352452	11554043	14362527	980616	2715057	89.34%
2	Untreated-2	39295796	15364770	17495732	1241430	2785415	90.08%
3	Tamoxifen-treated-1	33269977	8829978	16689260	1157647	3948253	84.97%
4	Tamoxifen-treated-2	26873058	9399729	12375253	920064	2374041	87.91%


### Differentially Expressed Genes (DEGs)

The cuffdiff and DESeq programs were used to determine the DEGs in tamoxifen-treated and untreated cells. The gene expression levels were considered statistically meaningful if *q*-values were <0.01. The data showed that from 4,710 genes with differential expression levels 2,713 genes were upregulated in tamoxifen-treated MCF-7 cells compared with controls (**Supplementary Table S2**). A total of 65 genes were apoptotic intermediates, especially in the Tp53 signaling pathway, and some of them including MAP2K2 and MAPK6 are involved in cell growth (**Supplementary Table S2**). In addition, down regulation of RARA, GREB and ESR1 (ER-alpha) genes was observed. These genes have key roles in classic Estrogen signaling pathways (**Supplementary Table S3**), thus, indicating that Tamoxifen can lead to apoptosis and cell death not only via modulation of the classic Estrogen signaling pathway but also by transcriptional activation of growth signaling pathways through GPR30 signaling axis, Next, the DEGs were subjected to functional annotation and qPCR verification.

### Gene Set Enrichment Analysis Using Gene Ontology (GO) Classification and KEGG Mapping

Using DAVID and GO functional enrichment analysis, we classified the DEGs data into three groups including biological process, cellular component, and molecular function. These results showed that 65 identified overexpressed genes belonged to the pro-apoptotic factors (*p*-value < 1.6E-05, *q*-value < 0.0007), of which 37 genes were involved in intrinsic apoptosis (*p*-value < 0.0002, *q*-value < 0.006) (**Supplementary Table S4**).

The KEGG database^1^ is designed to investigate the molecular interactions and signaling networks in cells. In order to identify the activated signaling pathway in tamoxifen-treated MCF-7 cells, the DEGs data were mapped to the KEGG, and 24 signaling pathways were identified (*q*-value < 0.01). The Tp53 signaling pathway was one of the pathways that was allocated to a large number of DEGs (*p*-value < 0.0003, *q*-value < 0.008). Based on the results in **Supplementary Table S5**, a total of 19 DEGs (CDKN1A; GADD45B; GADD45A; GORAB; TNFRSF10B; RCHY1; PPM1D; GADD45G; CASP8; ZMAT3; CASP3; SESN1; SESN2; FAS; CYCS; PMAIP1; MDM4; SFN; TP53) of the Tp53 signaling pathway were upregulated by tamoxifen stimulation, and could mediate cell apoptosis. On the other hand, the level of extrinsic apoptotic intermediates including SMAD3, NF-1, BCL-2, BOK, KIAA0141, ITGAV, JAK3, LMNA and RET were downregulated in tamoxifen-treated MCF-7 cells, which suggest that the Tp53 signaling pathway is the main pathway by which intrinsic apoptosis transduces signals in order to trigger cell death.

### Real-Time PCR Verification of DEGs

**FIGURE 7:**
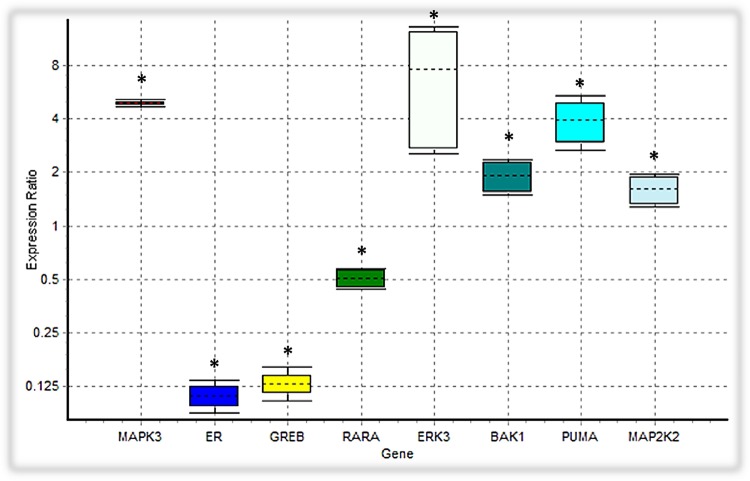
Analysis of the expression level of intended DEGs using qPCR. *Bak1*, *PUMA*, *ERK3* and *MAP2K2* genes were overexpressed in tamoxifen-treated MCF-7 cancer cells, while the expression level of *MAPK3* was not significantly changed. On the contrary, *ER (ESR1)*, *GREB* and *RARA* were depressed under tamoxifen induction. Each value is the mean ± SD of three separate experiments. The statistical significant differences are indicated with ^∗^ for *p* < 0.05.

In our ODE model, we showed that tamoxifen can engage the Ras/MAPK pathway through GPR30, triggering the activation of ERKs along with Tp53 mediated apoptosis pathway. Besides inducing the apoptosis intermediates, the expression level of the MAP2K2, CRAF and ERK3, which are involved in the MAPK signaling cascades, were also upregulated (**Figure 7**).

We verified the data obtained from RNA-seq analysis for 8 DEGs using the qPCR method (**Table 2**). As shown in **Table 2**, Bak1, PUMA, ERK3 and MAP2K2 genes were upregulated in MCF-7 cells incubated with tamoxifen. However, the MAPK3 expression level was not significantly altered. Also, the expression of genes such as ESR1(ER-alpha), GREB and RARA was decreased. Taken together, these observations were in line with RNA-seq results and confirmed the reliability of our ODE model, which indicated that Tamoxifen can also stimulate apoptotic cell death through increasing the magnitude of ERK activation and deregulation of cell growth intermediates.

**Table 2:** qPCR analysis of the desired genes.

Gene	Type	Expression	Standard error	95% C.I.	P(H1)	Result
*GAPDH*	REF	1.000				
*MAPK3*	TRG	4.942	4.771 – 5.119	4.731 – 5.161	0.162	
*ER (ESR1)*	TRG	0.110	0.092 – 0.131	0.089 – 0.135	0.000	DOWN
*GREB*	TRG	0.128	0.109 – 0.151	0.104 – 0.158	0.000	DOWN
*RARA*	TRG	0.498	0.435 – 0.570	0.435 – 0.570	0.000	DOWN
*ERK3*	TRG	5.836	2.637 – 12.928	2.578 – 13.215	0.000	UP
*BAK1*	TRG	1.879	1.520 – 2.323	1.508 – 2.342	0.000	UP
*PUMA*	TRG	3.811	2.816 – 5.171	2.698 – 5.386	0.000	UP
*MAP2K2*	TRG	1.575	1.283 – 1.933	1.276 – 1.943	0.000	UP


*P(H1), probability of alternate hypothesis that difference between sample and control groups is due only to chance. TRG, target; REF, reference.*

## Discussion

Estrogen is an important hormone in mammalian biology which exerts its effects on a diverse array of target tissues via genomic and non-genomic mechanisms (Filardo et al., 2002; Prossnitz and Maggiolini, 2009). It has been shown that GPR30 can mediate estradiol proliferative effects in thyroid (Vivacqua et al., 2006a), endometrial (Vivacqua et al., 2006b), ovarian (Albanito et al., 2007) and GPR30-positive SKBr3 breast cancer cell lines (Albanito et al., 2008). In this work we utilize the heat-inactivated FBS to diminish the estrogen concentration. There has been some evidence regarding the ability of GPR30 to interact with low levels of estrogens leading to PI3K and MAPK activation (Filardo et al., 2000, 2002). Further, it has been shown that GPR30 is required for estrogen-induced activation of the MAPKs, ERK-1 and ERK-2. Additionally, estrogen-mediated ERK1/2 activation through GPR30 pathway is transient and rapidly returning to basal levels 10–15 min after exposure to estrogen (Filardo et al., 2002). It is also worth pointing out that, we have compared the gene expression profile between the tamoxifen-treated and untreated cells, thus, the interference effects of the residual estrogen for each group should be similar.

Tamoxifen, a potent anticancer agent known to interrupt the enhanced estrogen activity of malignant mammary gland cells, is used in women with breast cancer or at risk of developing it (Larosche et al., 2007; Zheng et al., 2007). Tamoxifen treatment displayed inhibitory effects on HeLa cell multiplication at lower concentrations and toxicity at higher concentrations and longer treatment durations. Cytological observations also showed nuclear condensation, cell shrinkage, multinucleation, and apoptotic bodies (Majumdar et al., 2001). Moreover, the tamoxifen induced reduction of cell viability in HepG2 cells depends on drug concentration and cell density and is due to cytostatic and cytocide effects (Brandt et al., 2004). It was also shown that tamoxifen (50, 100, 200, or 250 μM) inhibited CPT-I activity by 38, 43, 58, and 64%, respectively (Larosche et al., 2007).

The GPR30 receptor is a membrane ER-like protein that actives intracellular signaling mediators, such as ERKs (Filardo et al., 2000; Migliaccio et al., 2010; Ge et al., 2014; Wei et al., 2014). Recently, many investigators have reported that GPCRs such as GPR30, enhance the MMPs mediated HB-EGF protein release, and initiate the EGFR signaling. Also, the Gβγ complex, formed in response to GPCRs activation, stimulates the Src and Shc function and activate RTKs (Luttrell et al., 1999; Filardo et al., 2000; Filardo, 2002; Prossnitz and Maggiolini, 2009). Apart from the RTKs, GPCRs also have the ability to activate ERKs in an alternative manner by stimulating mononuclear GTPases such as Ras or Rap proteins (Belcheva and Coscia, 2002; Bouschet et al., 2003; New and Wong, 2007).

The activation of ERKs is associated with many classical apoptotic interactions such as activation of Caspase-3, PARP-1 cleavage, accumulation of phosphatidylserine in the membrane, and aggregation and fragmentation of genomic DNA (Wang et al., 2000; Alexia et al., 2004; Lee et al., 2005, 2006; Liu et al., 2008). Considering the types of cells and the undertaken damage, the activity of the Ras/Raf/ERK signaling pathway may lead to the release of cytochrome c from mitochondria and activation of caspase-9, or alternatively, stimulate extrinsic apoptotic pathway that relies on the caspase-8 activity (Wang et al., 2000; Nesterov et al., 2004; Schweyer et al., 2004; Zhang et al., 2004; Basu and Tu, 2005; Wu et al., 2005). The activated ERKs can be located in the mitochondrial membrane, and by disrupting could cause the release of cytochrome c to the cytoplasm (Nowak, 2002; Nowak et al., 2006; Zhuang et al., 2007). Also, ERK-dependent over-activation of Tp53 protein, leads to phosphorylation of serine 15 in p53, inhibits its interaction with Mdm2 resulting in stability and accumulation of Tp53 (Persons et al., 2000; She et al., 2000; Yeh et al., 2004).

Bcl-2 family proteins have also an important role in the cytochrome c excretion and induction of the inherited apoptosis. The activation of MEK/ERKs increases the expression of pro-apoptotic members of the Bcl-2 family including Bax, PUMA and Bak. On the contrary, it depresses the anti-apoptotic members of the Bcl-2 family, such as Bcl-2 itself and Bcl-XL (Li et al., 2007; Liu et al., 2008; Panaretakis et al., 2008). Additionally, the activity of Bcl-2 family is significantly regulated by the Tp53 protein. ERK can induce the phosphorylation of threonine 55 in Tp53, enhance its binding to the DNA, and down regulate Bcl-2 expression (Persons et al., 2000; She et al., 2000).

Many evidence suggest that estrogen signaling could be mediated by the GPR30 as well as ER receptors. Tamoxifen, an agonist for GPR30 receptors, is widely used as a selective estrogen receptor (SERM) in treating hormone-dependent breast cancer (Thomas et al., 2005; Jordan, 2006; Prossnitz et al., 2008). We simulated the activity of the GPR30/PI3K/MAPK signaling pathway in the onset of tamoxifen induced apoptosis within MCF-7 cells. Our experiments depicted that the expression level of 4,710 unique genes was altered in the presence of tamoxifen. The GO analysis showed that many of the marked genes play an active role in cell apoptosis pathways. For example, 65 genes were associated with the apoptotic signaling pathway (GO:0097190). Also, 37 genes related to intrinsic apoptotic signaling pathway (GO:0097193) were upregulated. On the other hand, 22 genes have a positive role in cell growth (GO:0030307), which demonstrates the impact of deregulated growth signaling pathways on initiation of apoptosis in cells incubated with tamoxifen (**Supplementary Table S4**). However, this theory needs further examinations. The KEGG analysis showed that many signaling pathways, including the p53 pathway, were significantly affected with tamoxifen treatment. In addition to a significant increase in the expression levels of the Tp53 signaling intermediates, such as Bak and PUMA, the expression of ERK3 and MAP2K2 genes, involved in the MAPK signaling pathways, were also increased. Furthermore, decreased expression of genes such as ESR1 (ER-alpha), RARA and GREB (**Figure 7**) suggests that the cytotoxic effects of tamoxifen were not only mediated by disabling the ER and some related factors, but most likely mediated through the GPR30/PI3K/MAPKs interactions, ERK activation, and finally the intrinsic apoptosis mediated by Tp53 (**Supplementary Table S2**).

In this study, a mathematical model was developed for the first time to assess the cross-talk between the GPR30, EGFR, PI3K and STAT signaling pathways in the presence or absence of tamoxifen (**Supplementary Table S1**). These four biological processes are considered as main pathways during cell proliferation process. Thus, any alterations in their expression or function can lead to cell death. Based on the RNA-seq analysis, the expression level of many MAPK pathway mediators, including MAP2K2, Myc, MAPK6, JUN, and Raf-1, were increased in the treated cells (**Supplementary Tables S2**, **S6**). Also, the designed model of these four pathways showed an enhancement in the level of activating phosphorylation of MAPKs by tamoxifen stimulation.

## Conclusion

In summary, this study showed that the apoptotic effects of tamoxifen on MCF-7 cells were mediated through activation of important signaling pathways including Tp53 and MAPKs, and induction of intrinsic apoptosis. Our simulation and transcriptomics analysis also showed that GPR30 activation may have a potential role in the activation of MAPK pathway and it is predicted that GPR30/PI3K/MAPK/STAT axis may be crucial for induction of apoptosis in MCF-7 cell lines. Based on these investigations, we claimed that GPER pathway might be involved in toxicity of tamoxifen in ER positive MCF-7 cells. In addition to enhancing our systematic understanding of the efficacy of tamoxifen in treating breast cancer, this study introduced the signaling pathways that impact the progression or prevention of the breast cancer development and metastasis.

## Author Contributions

MR: designed and performed the experiments, prepared the figures and/or tables, analyzed the data, performed bioinformatic analyses, and wrote the paper. SS: supervised the research, conceived and designed experiments, wrote the paper, and reviewed drafts of the paper. JC, NS, and GB reviewed drafts of the paper and technical support. All authors discussed the results and contributed to the final manuscript.

## Conflict of Interest Statement

The authors declare that the research was conducted in the absence of any commercial or financial relationships that could be construed as a potential conflict of interest.

## Footnotes

1 http://www.genome.jp/kegg

## Supplementary Material

TABLE S1 List of model reactions for simulated pathways.

TABLE S2 Summary of RNA sequence analysis for up-regulated genes.

TABLE S3 Summary of RNA sequence analysis for down-regulated genes.

TABLE S4 Gene ontology (GO) analysis of differentially expressed genes.

TABLE S5 Gene set enrichment analysis using KEGG mapping.

TABLE S6 List of up-regulated isoforms.

## Abbreviations

AKT: AKT serine/threonine kinase
BAK: BCL2 Antagonist/Killer 1
BAX: BCL2 Associated X
CASP3: caspase 3
CASP8: caspase 8
CDKN1A: cyclin dependent kinase inhibitor 1A
CYCS: cytochrome C, somatic
DAG: diacylglycerol
EGF: epidermal growth factor
FAS: Fas Cell Surface Death Receptor
GADD45A: growth arrest and DNA damage inducible alpha
GADD45B: growth arrest and DNA damage inducible beta
GADD45G: growth arrest and DNA damage inducible gamma
GORAB: golgin, RAB6 interacting
Grb-2: growth factor receptor bound protein 2
GREB: growth regulation by estrogen in breast cancer
IP3: inositol triphosphate
ITGAV: integrin subunit alpha V
JAK: Janus kinase
LMNA: Lamin A/C
NF1: neurofibromin 1
PARP: poly(ADP-ribose) polymerase
PI3K: phosphoinositide-3-kinase
PKC: protein kinase C
PLC: phospholipase C
PMAIP1: phorbol-12-myristate-13-acetate-induced protein 1
PPM1D: protein phosphatase, Mg2+/Mn2+ dependent 1D
PTEN: phosphatase and tensin homolog
PUMA: P53 up-regulated modulator of apoptosis
RARA: retinoic acid receptor alpha
RCHY1: ring finger and CHY zinc finger domain containing 1
RTK: receptor tyrosine kinase
SESN: sestrin
SFN: stratifin
STAT: signal transducer and activator of transcription
TNFRSF10B: TNF receptor superfamily member 10b
ZMAT3: zinc finger matrin-type 3
