# Supplementary material for: Tamoxifen-Induced Apoptosis of MCF-7 Cells via GPR30/PI3K/MAPKs Interactions: Verification by ODE Modeling and RNA Sequencing
Source: Front Physiol. 2018 Jul 11;9:907. doi: 10.3389/fphys.2018.00907 (PMC6050429; doi:10.3389/fphys.2018.00907)
Supplement: TABLE S1 — List of model reactions for simulated pathways. [file Data_Sheet_1.DOCX]

**Supplementary Table 1**

1. **Model reactions**

Note: Michaelis Menten constants are given in µM, first order rate constants in s^-1^ and second order rate constants in µM^-1^ s^-1^

| reactions | | parameters | | References |
| --- | --- | --- | --- | --- |
| r1 | EGF + EGFR <-> [EGF-EGFR] | k1= 100 | kr1= 0.0038 | ([Schoeberl et al., 2002](#_ENREF_10); [Yamada et al., 2004](#_ENREF_13)) |
| r2 | [EGF-EGFR] + [EGF-EGFR] <-> [EGF-EGFR2] | k2= 10 | kr2= 0.02 | ([Yamada et al., 2004](#_ENREF_13); [Sasagawa et al., 2005](#_ENREF_9)) |
| r3 | [EGF-EGFR2] -> [pEGF-EGFR2] | k3=2.014 |  | ([Yamada et al., 2004](#_ENREF_13)) |
| r6 | [pEGF-EGFR2] + SHP2 <-> [pEGF-EGFR2-SHP2] | k6=3.114 | kr6=0.2 | ([Yamada et al., 2004](#_ENREF_13)) |
| r7 | [pEGF-EGFR2-SHP2] -> [EGF-EGFR2] + SHP2 | k7=2.661 |  | ([Yamada et al., 2004](#_ENREF_13)) |
| r8 | [pEGF-EGFR2] + Shc <-> [pEGF-EGFR2-Shc] | k8=90 | kr8=0.6 | ([Yamada et al., 2004](#_ENREF_13)) |
| r9 | [pEGF-EGFR2-Shc] -> [pEGF-EGFR2-pShc] | k9=0.5838 |  | ([Yamada et al., 2004](#_ENREF_13)) |
| r10 | [pEGF-EGFR2-pShc] <-> [pEGF-EGFR2] + pShc | k10=4.481 | kr10=0.3 | ([Yamada et al., 2004](#_ENREF_13)) |
| r11 | pShc + SHP2 <-> [pShc-SHP2] | k11=3.114 | kr11=0.2 | ([Yamada et al., 2004](#_ENREF_13)) |
| r12 | [pShc-SHP2] -> Shc + SHP2 | k12=0.2661 |  | ([Yamada et al., 2004](#_ENREF_13)) |
| r13 | pShc -> Shc | k13=0.005 |  | ([Yamada et al., 2004](#_ENREF_13)) |
| r18 | [pEGF-EGFR2-pShc] + Grb2 <-> [pEGF-EGFR2-pShc-Grb2] | k18=3 | kr18=0.1 | ([Kholodenko et al., 1999](#_ENREF_6)) |
| r19 | [pEGF-EGFR2-pShc-Grb2] + SHP2 <-> [pEGF-EGFR2-pShc-Grb2-SHP2] | k19=10 | kr19=1 | ([Yamada et al., 2004](#_ENREF_13)) |
| r20 | [pEGF-EGFR2-pShc-Grb2-SHP2] -> [EGF-EGFR2] + pShc + Grb2 + SHP2 | k20=2.661 |  | ([Yamada et al., 2004](#_ENREF_13)) |
| r23 | [pEGF-EGFR2-pShc-Grb2] + SOS <-> [pEGF-EGFR2-pShc-Grb2-SOS] | k23=10 | kr23=0.0214 | ([Kholodenko et al., 1999](#_ENREF_6)) |
| r26 | Grb2 + SOS <-> [Grb2-SOS] | k26=0.1 | kr26=0.0015 | ([Kholodenko et al., 1999](#_ENREF_6)) |
| r27 | [pEGF-EGFR2-pShc] + [Grb2-SOS] <-> [pEGF-EGFR2-pShc-Grb2-SOS] | k27=10 | kr27=0.045 | ([Kholodenko et al., 1999](#_ENREF_6)) |
| r28 | [pEGF-EGFR2] + Grb2 <-> [pEGF-EGFR2-Grb2] | k28=3 | kr28=0.05 | ([Kholodenko et al., 1999](#_ENREF_6)) |
| r29 | [pEGF-EGFR2-Grb2] + SHP2 <-> [pEGF-EGFR2-Grb2-SHP2] | k29=10 | kr29=1 | ([Yamada et al., 2004](#_ENREF_13)) |
| r30 | [pEGF-EGFR2-Grb2-SHP2] -> [EGF-EGFR2] + Grb2 + SHP2 | k30=2.661 |  | ([Yamada et al., 2004](#_ENREF_13)) |
| r31 | [pEGF-EGFR2-Grb2] + SOS <-> [pEGF-EGFR2-Grb2-SOS] | k31=10 | kr31=0.06 | ([Kholodenko et al., 1999](#_ENREF_6)) |
| r32 | [pEGF-EGFR2] + [Grb2-SOS] <-> [pEGF-EGFR2-Grb2-SOS] | k32=2.734 | kr32=0.025 | ([Yamada et al., 2004](#_ENREF_13)) |
| r35 | [pEGF-EGFR2-pShc-Grb2-SOS] + [Ras-GDP] <-> [pEGF-EGFR2-pShc-Grb2-SOS-Ras-GDP] | k35=202.9 | kr35=0.18 | ([Yamada et al., 2004](#_ENREF_13)) |
| r36 | [pEGF-EGFR2-pShc-Grb2-SOS-Ras-GDP] -> [pEGF-EGFR2-pShc-Grb2-SOS] + [Ras-GTP] | k36=0.1434 |  | ([Yamada et al., 2004](#_ENREF_13)) |
| r37 | [pEGF-EGFR2-Grb2-SOS] + [Ras-GDP] <-> [pEGF-EGFR2-Grb2-SOS-Ras-GDP] | k37=202.9 | kr37=0.18 | ([Yamada et al., 2004](#_ENREF_13)) |
| r38 | [pEGF-EGFR2-Grb2-SOS-Ras-GDP] -> [pEGF-EGFR2-Grb2-SOS] + [Ras-GTP] | k38=0.1434 |  | ([Yamada et al., 2004](#_ENREF_13)) |
| r39 | [Ras-GTP] -> [Ras-GDP] | k39=0.000167 |  | ([Yamada et al., 2004](#_ENREF_13)) |
| r40 | [Ras-GTP] + [Ras-GAP] <-> [Ras-GTP-Ras-GAP] | k40=2.854 | kr40=0.96 | ([Yamada et al., 2004](#_ENREF_13)) |
| r41 | [Ras-GTP-Ras-GAP] -> [Ras-GDP] + [Ras-GAP] | k41=7.76 |  | ([Yamada et al., 2004](#_ENREF_13)) |
| r42 | [pEGF-EGFR2] + [Ras-GAP] <-> [pEGF-EGFR2-Ras-GAP] | k42=0.1 | kr42=0.01 | ([Yamada et al., 2004](#_ENREF_13)) |
| r43 | [pEGF-EGFR2-Ras-GAP] + [Ras-GTP] <-> [pEGF-EGFR2-Ras-GAP-Ras-GTP] | k43=2.845 | kr43=0.96 | ([Yamada et al., 2004](#_ENREF_13)) |
| r44 | [pEGF-EGFR2-Ras-GAP-Ras-GTP] -> [pEGF-EGFR2-Ras-GAP] + [Ras-GDP] | k44=7.76 |  | ([Yamada et al., 2004](#_ENREF_13)) |
| r45 | [pEGF-EGFR2-Ras-GAP] + SHP2 <-> [pEGF-EGFR2-Ras-GAP-SHP2] | k45=3.114 | kr45=0.2 | ([Yamada et al., 2004](#_ENREF_13)) |
| r46 | [pEGF-EGFR2-Ras-GAP-SHP2] -> [EGF-EGFR2] + [Ras-GAP] + SHP2 | k46=2.661 |  | ([Yamada et al., 2004](#_ENREF_13)) |
| r47 | Raf1 + [Ras-GTP] <-> [Raf1-Ras-GTP] | k47=1.75 | kr47=0.05 | ([Yamada et al., 2004](#_ENREF_13)) |
| r48 | [Raf1-Ras-GTP] -> Raf1active + [Ras-GTP] | k48=0.7624 |  | ([Yamada et al., 2004](#_ENREF_13)) |
| r49 | Raf1active + MEK <-> [Raf1active-MEK] | k49=4 | kr49=0.01833 | ([Huang and Ferrell, 1996](#_ENREF_5); [Schoeberl et al., 2002](#_ENREF_10); [Ung et al., 2008](#_ENREF_11)) |
| r50 | [Raf1active-MEK] -> Raf1active + pMEK | k50=3.5 |  | ([Huang and Ferrell, 1996](#_ENREF_5); [Schoeberl et al., 2002](#_ENREF_10); [Ung et al., 2008](#_ENREF_11)) |
| r51 | Raf1active + pMEK <-> [Raf1active-pMEK] | k51=4 | kr51=0.01833 | ([Huang and Ferrell, 1996](#_ENREF_5); [Schoeberl et al., 2002](#_ENREF_10); [Ung et al., 2008](#_ENREF_11)) |
| r52 | [Raf1active-pMEK] -> Raf1active + ppMEK | k52=2.9 |  | ([Huang and Ferrell, 1996](#_ENREF_5); [Schoeberl et al., 2002](#_ENREF_10); [Ung et al., 2008](#_ENREF_11)) |
| r53 | ppMEK + ERK <-> [ppMEK-ERK] | k53=3 | kr53=0.033 | ([Huang and Ferrell, 1996](#_ENREF_5); [Schoeberl et al., 2002](#_ENREF_10); [Ung et al., 2008](#_ENREF_11)) |
| r54 | [ppMEK-ERK] -> ppMEK + pERK | k54=16 |  | ([Huang and Ferrell, 1996](#_ENREF_5); [Schoeberl et al., 2002](#_ENREF_10); [Ung et al., 2008](#_ENREF_11)) |
| r55 | ppMEK + pERK <-> [ppMEK-pERK] | k55=3 | kr55=0.033 | ([Huang and Ferrell, 1996](#_ENREF_5); [Schoeberl et al., 2002](#_ENREF_10); [Ung et al., 2008](#_ENREF_11)) |
| r56 | [ppMEK-pERK] -> ppMEK + ppERK | k56=5.7 |  | ([Huang and Ferrell, 1996](#_ENREF_5); [Schoeberl et al., 2002](#_ENREF_10); [Ung et al., 2008](#_ENREF_11)) |
| r57 | Raf1active + Pase <-> [Raf1active-Pase] | k57=71.7 | kr57=0.2 | ([Huang and Ferrell, 1996](#_ENREF_5); [Schoeberl et al., 2002](#_ENREF_10); [Ung et al., 2008](#_ENREF_11)) |
| r58 | [Raf1active-Pase] -> Raf1 + Pase | k58=1 |  | ([Huang and Ferrell, 1996](#_ENREF_5); [Schoeberl et al., 2002](#_ENREF_10); [Ung et al., 2008](#_ENREF_11)) |
| r59 | ppMEK + Pase2 <-> [ppMEK-Pase2] | k59=14.3 | kr59=0.8 | ([Huang and Ferrell, 1996](#_ENREF_5); [Schoeberl et al., 2002](#_ENREF_10); [Ung et al., 2008](#_ENREF_11)) |
| r60 | [ppMEK-Pase2] -> pMEK + Pase2 | k60=0.058 |  | ([Huang and Ferrell, 1996](#_ENREF_5); [Schoeberl et al., 2002](#_ENREF_10); [Ung et al., 2008](#_ENREF_11)) |
| r61 | pMEK + Pase2 <-> [pMEK-Pase2] | k61=0.25 | kr61=0.5 | ([Huang and Ferrell, 1996](#_ENREF_5); [Schoeberl et al., 2002](#_ENREF_10); [Ung et al., 2008](#_ENREF_11)) |
| r62 | [pMEK-Pase2] -> MEK + Pase2 | k62=0.58 |  | ([Huang and Ferrell, 1996](#_ENREF_5); [Schoeberl et al., 2002](#_ENREF_10); [Ung et al., 2008](#_ENREF_11)) |
| r63 | ppERK + Pase3 <-> [ppERK-Pase3] | k63=7 | kr63=0.6 | ([Huang and Ferrell, 1996](#_ENREF_5); [Schoeberl et al., 2002](#_ENREF_10); [Ung et al., 2008](#_ENREF_11)) |
| r64 | [ppERK-Pase3] -> pERK + Pase3 | k64=0.27 |  | ([Huang and Ferrell, 1996](#_ENREF_5); [Schoeberl et al., 2002](#_ENREF_10); [Ung et al., 2008](#_ENREF_11)) |
| r65 | pERK + Pase3 <-> [pERK-Pase3] | k65=5 | kr65=0.5 | ([Huang and Ferrell, 1996](#_ENREF_5); [Schoeberl et al., 2002](#_ENREF_10); [Ung et al., 2008](#_ENREF_11)) |
| r66 | [pERK-Pase3] -> ERK + Pase3 | k66=0.3 |  | ([Huang and Ferrell, 1996](#_ENREF_5); [Schoeberl et al., 2002](#_ENREF_10); [Ung et al., 2008](#_ENREF_11)) |
| r67 | ppERK + [pEGF-EGFR2-pShc-Grb2-SOS] <-> [ppERK-pEGF-EGFR2-pShc-Grb2-SOS] | k67=8.898 | kr67=1 | ([Yamada et al., 2004](#_ENREF_13)) |
| r68 | [ppERK-pEGF-EGFR2-pShc-Grb2-SOS] -> ppERK + [pEGF-EGFR2] + pShc + Grb2 + pSOS | k68=0.0426 |  | ([Yamada et al., 2004](#_ENREF_13)) |
| r69 | ppERK + [pEGF-EGFR2-Grb2-SOS] <-> [ppERK-pEGF-EGFR2-Grb2-SOS] | k69=8.898 | kr69=1 | ([Yamada et al., 2004](#_ENREF_13)) |
| r70 | [ppERK-pEGF-EGFR2-Grb2-SOS] -> ppERK + [pEGF-EGFR2] + Grb2 + pSOS | k70=0.0426 |  | ([Yamada et al., 2004](#_ENREF_13)) |
| r71 | pSOS -> SOS | k71=0.002 |  | ([Sasagawa et al., 2005](#_ENREF_9)) |
| r72 | ProEGFR -> EGFR | k72=0.005 |  | ([Yamada et al., 2004](#_ENREF_13)) |
| r73 | [pEGF-EGFR2-pShc-Grb2-SOS] + cbl <-> [pEGF-EGFR2-pShc-Grb2-SOS-cbl] | k73=0.5 | kr73=0.005 | ([Yamada et al., 2004](#_ENREF_13)) |
| r74 | [pEGF-EGFR2-pShc-Grb2-SOS-cbl] + EPn <-> [pEGF-EGFR2-pShc-Grb2-SOS-cbl-EPn] | k74=5 | kr74=0.1 | ([Yamada et al., 2004](#_ENREF_13)) |
| r75 | [pEGF-EGFR2-pShc-Grb2-SOS-cbl-EPn] -> cbl + [Grb2-SOS] + EPn + pShc | k75=0.001 |  | ([Yamada et al., 2004](#_ENREF_13)) |
| r76 | [pEGF-EGFR2-Grb2-SOS] + cbl <-> [pEGF-EGFR2-Grb2-SOS-cbl] | k76=0.5 | kr76=0.005 | ([Yamada et al., 2004](#_ENREF_13)) |
| r77 | [pEGF-EGFR2-Grb2-SOS-cbl] + EPn <-> [pEGF-EGFR2-Grb2-SOS-cbl-EPn] | k77=5 | kr77=0.1 | ([Yamada et al., 2004](#_ENREF_13)) |
| r78 | [pEGF-EGFR2-Grb2-SOS-cbl-EPn] -> cbl + [Grb2-SOS] + EPn | k78=0.001 |  | ([Yamada et al., 2004](#_ENREF_13)) |
| r79 | [pEGF-EGFR2] + cbl <-> [pEGF-EGFR2-cbl] | k79=0.5 | kr79=0.005 | ([Yamada et al., 2004](#_ENREF_13); [Sasagawa et al., 2005](#_ENREF_9)) |
| r80 | [pEGF-EGFR2-cbl] + EPn <-> [pEGF-EGFR2-cbl-EPn] | k80=5 | kr80=0.1 | ([Yamada et al., 2004](#_ENREF_13); [Sasagawa et al., 2005](#_ENREF_9)) |
| r81 | [pEGF-EGFR2-cbl-EPn] -> cbl + EPn | k81=0.001 |  | ([Yamada et al., 2004](#_ENREF_13); [Sasagawa et al., 2005](#_ENREF_9)) |
| r82 | [pEGF-EGFR2] + PI3K <-> [pEGF-EGFR2-PI3K] | k82=14 | kr82=0.1743 | ([Kiyatkin et al., 2006](#_ENREF_7); [Ung et al., 2008](#_ENREF_11)) |
| r83 | [pEGF-EGFR2-PI3K] <-> [pEGF-EGFR2-pPI3K] | k83=33.72 | kr83=0.000337 | ([Kiyatkin et al., 2006](#_ENREF_7); [Ung et al., 2008](#_ENREF_11)) |
| r84 | [pEGF-EGFR2-PI3K] <-> [pEGF-EGFR2] + pPI3K | k84=0.09 | kr84=0.1764 | ([Kiyatkin et al., 2006](#_ENREF_7)) |
| r85 | TP4 + pPI3K <-> [TP4-pPI3K] | k85=1 | kr85=0.038 | ([Kiyatkin et al., 2006](#_ENREF_7)) |
| r86 | [TP4-pPI3K] -> [TP4-PI3K] | k86=0.595 |  | ([Kiyatkin et al., 2006](#_ENREF_7)) |
| r87 | [TP4-PI3K] <-> TP4 + PI3K | k87=4.7E-06 | kr87=2.3E-06 | ([Kiyatkin et al., 2006](#_ENREF_7)) |
| r88 | pPI3K + PIP2 <-> [pPI3K-PIP2] | k88=25 | kr88=3.5 | ([Kiyatkin et al., 2006](#_ENREF_7)) |
| r89 | [pPI3K-PIP2] -> pPI3K + PIP3 | k89=25 |  | ([Kiyatkin et al., 2006](#_ENREF_7)) |
| r90 | Akt + PIP3 <-> Aktm | k90=10 | kr90=3 | ([Kiyatkin et al., 2006](#_ENREF_7)) |
| r91 | Aktm + PDK1 <-> [Aktm-PDK1] | k91=10 | kr91=1 | ([Kiyatkin et al., 2006](#_ENREF_7)) |
| r92 | [Aktm-PDK1] -> [pAktm-PDK1] | k92=10 |  | ([Kiyatkin et al., 2006](#_ENREF_7)) |
| r93 | [pAktm-PDK1] <-> pAktm + PDK1 | k93=0.1 | kr93=0.005 | ([Kiyatkin et al., 2006](#_ENREF_7)) |
| r94 | pAktm <-> pAkt + PIP3 | k94=1 | kr94=0.001 | ([Kiyatkin et al., 2006](#_ENREF_7)) |
| r95 | [pAkt-Takt] -> [Akt-Takt] | k95=0.05 |  | ([Kiyatkin et al., 2006](#_ENREF_7)) |
| r96 | [Akt-Takt] <-> Akt + Takt | k96=0.001 | kr96=0.001 | ([Kiyatkin et al., 2006](#_ENREF_7)) |
| r97 | pAkt + Takt <-> [pAkt-Takt] | k97=10 | kr97=1 | ([Kiyatkin et al., 2006](#_ENREF_7)) |
| r98 | pAktm + Takt <-> [pAktm-Takt] | k98=10 | kr98=1 | ([Kiyatkin et al., 2006](#_ENREF_7)) |
| r99 | [pAktm-Takt] -> [Aktm-Takt] | k99=0.05 |  | ([Kiyatkin et al., 2006](#_ENREF_7)) |
| r100 | [Aktm-Takt] <-> Aktm + Takt | k100=0.001 | kr100=0.001 | ([Kiyatkin et al., 2006](#_ENREF_7)) |
| r101 | [pAktm-PDK1] + Takt <-> [pAktm-PDK1-Takt] | k101=10 | kr101=1 | ([Kiyatkin et al., 2006](#_ENREF_7)) |
| r102 | [pAktm-PDK1-Takt] -> [Aktm-PDK1-Takt] | k102=0.05 |  | ([Kiyatkin et al., 2006](#_ENREF_7)) |
| r103 | [Aktm-PDK1-Takt] <-> [Aktm-PDK1] + Takt | k103=0.001 | kr103=0.001 | ([Kiyatkin et al., 2006](#_ENREF_7)) |
| r104 | Raf1active + pAkt_total -> pRaf1active + pAkt_total | Kon= 0.1 | km= 0.2 | ([Kiyatkin et al., 2006](#_ENREF_7)) |
| r105 | pRaf1active -> Raf1active | k105=1 |  | ([Kiyatkin et al., 2006](#_ENREF_7)) |
| 106 | [pEGF-EGFR2] + STAT3c <-> [pEGF-EGFR2-STAT3c] | k106=5.5 | kr106=11.74 | ([Hsieh et al., 2010](#_ENREF_4)),estimation |
| 107 | [pEGF-EGFR2-STAT3c] -> [pEGF-EGFR2] + pSTAT3c | k107=0.4 |  | ([Yamada et al., 2003](#_ENREF_12)) |
| 108 | [pEGF-EGFR2] + pSTAT3c <-> [pEGF-EGFR2-pSTAT3c] | k108=5 | kr108=0.5 | ([Yamada et al., 2003](#_ENREF_12)) |
| 109 | pSTAT3c + PP1 <-> [pSTAT3c-PP1] | k109=1 | kr109=0.2 | ([Yamada et al., 2003](#_ENREF_12)) |
| 110 | [pSTAT3c-PP1] -> STAT3c + PP1 | k110=0.003 |  | ([Yamada et al., 2003](#_ENREF_12)) |
| 111 | pSTAT3c + pSTAT3c <-> [pSTAT3c-pSTAT3c] | k111=20 | kr111=0.1 | ([Yamada et al., 2003](#_ENREF_12)) |
| 112 | [pSTAT3c-pSTAT3c] + PP1 <-> [pSTAT3c-pSTAT3c-PP1] | k112=1 | kr112=0.2 | ([Yamada et al., 2003](#_ENREF_12)) |
| 113 | [pSTAT3c-pSTAT3c-PP1] -> [STAT3c-pSTAT3c] + PP1 | k113=0.003 |  | ([Yamada et al., 2003](#_ENREF_12)) |
| 114 | STAT3c + pSTAT3c <-> [STAT3c-pSTAT3c] | k114=0.0002 | kr114=0.2 | ([Yamada et al., 2003](#_ENREF_12)) |
| 115 | [pSTAT3c-pSTAT3c] -> [pSTAT3n-pSTAT3n] | k115=0.005 |  | ([Yamada et al., 2003](#_ENREF_12)) |
| 116 | pSTAT3n + pSTAT3n <-> [pSTAT3n-pSTAT3n] | k116=20 | kr116=0.1 | ([Yamada et al., 2003](#_ENREF_12)) |
| 117 | [pSTAT3n-pSTAT3n] + PP2 <-> [pSTAT3n-pSTAT3n-PP2] | k117=1 | kr117=0.2 | ([Yamada et al., 2003](#_ENREF_12)) |
| 118 | [pSTAT3n-pSTAT3n-PP2] -> [STAT3n-pSTAT3n] + PP2 | k118=0.005 |  | ([Yamada et al., 2003](#_ENREF_12)) |
| 119 | STAT3n + pSTAT3n <-> [STAT3n-pSTAT3n] | k119=0.0002 | kr119=0.2 | ([Yamada et al., 2003](#_ENREF_12)) |
| 120 | pSTAT3n + PP2 <-> [pSTAT3n-PP2] | k120=1 | kr120=0.2 | ([Yamada et al., 2003](#_ENREF_12)) |
| 121 | [pSTAT3n-PP2] -> STAT3n + PP2 | k121=0.005 |  | ([Yamada et al., 2003](#_ENREF_12)) |
| 122 | STAT3n -> STAT3c | k122=0.05 |  | ([Yamada et al., 2003](#_ENREF_12)) |
| r123 | PIP3 -> PIP2 | k123=17 |  | ([Yamada et al., 2003](#_ENREF_12)) |
| 124 | [pEGF-EGFR2-STAT3c] + cbl <-> [pEGF-EGFR2-STAT3c-cbl] | k124=0.5 | kr124=0.005 | ([Yamada et al., 2004](#_ENREF_13)), estimation |
| 125 | [pEGF-EGFR2-STAT3c-cbl] + EPn <-> [pEGF-EGFR2-STAT3c-cbl-EPn] | k125=5 | kr125=0.1 | ([Yamada et al., 2004](#_ENREF_13)) , estimation |
| 126 | [pEGF-EGFR2-STAT3c-cbl-EPn] -> STAT3c + cbl + EPn | k126=0.001 |  | ([Yamada et al., 2004](#_ENREF_13)) , estimation |
| 127 | [pEGF-EGFR2-PI3K] + cbl <-> [pEGF-EGFR2-PI3K-cbl] | k127=0.5 | kr127=0.005 | ([Yamada et al., 2004](#_ENREF_13)) , estimation |
| 128 | [pEGF-EGFR2-PI3K-cbl] + EPn <-> [pEGF-EGFR2-PI3K-cbl-EPn] | k128=5 | kr128=0.1 | ([Yamada et al., 2004](#_ENREF_13)) , estimation |
| 129 | [pEGF-EGFR2-PI3K-cbl-EPn] -> PI3K + EPn | k129=0.001 |  | ([Yamada et al., 2004](#_ENREF_13)) , estimation |
| 130 | TMX + GPR30 <-> [TMX-GPR30] | k130= 4500 | kr130= 0.0015 | ([Rich et al., 2002](#_ENREF_8))  estimation |
| 131 | Gq-trimer -> [Ga-GTP] | k131= 3.11E-4 |  | ([Heitzler et al., 2012](#_ENREF_3)) |
| 132 | [Ga-GTP] -> [Gq-trimer] | k132= 5.099 |  | ([Heitzler et al., 2012](#_ENREF_3)) |
| 133 | [TMX-GPR30] + [Gq-trimer] -> [Ga-GTP] + [TMX-GPR30] | k133= 7.6 |  | ([Heitzler et al., 2012](#_ENREF_3)) |
| 134 | [TMX-GPR30] + [GRK2/3] -> [TMX-GPR30-p] + [GRK2/3] | k134= 2.27 |  | ([Heitzler et al., 2012](#_ENREF_3)) |
| 135 | [TMX-GPR30-p] + barr1 -> [TMX-barr1-GPR30-p] | k135= 2.61 |  | ([Heitzler et al., 2012](#_ENREF_3)) |
| 136 | [TMX-barr1-GPR30-p] -> [TMX-GPR30-p] + barr1 | k136= 0.0060 |  | ([Heitzler et al., 2012](#_ENREF_3)) |
| 137 | [TMX-barr1-GPR30-p] -> barr1 + [TMX-GPR30] | k137= 6.54E-5 |  | ([Heitzler et al., 2012](#_ENREF_3)) |
| 138 | [TMX-GPR30-p] + barr2 -> [TMX-barr2-GPR30-p] | k138= 2.59 |  | ([Heitzler et al., 2012](#_ENREF_3)) |
| 139 | [TMX-barr2-GPR30-p] -> [TMX-GPR30-p] + barr2 | k139= 0.031 |  | ([Heitzler et al., 2012](#_ENREF_3)) |
| 140 | [TMX-barr2-GPR30-p] -> barr2 + [TMX-GPR30] | k140= 0.072 |  | ([Heitzler et al., 2012](#_ENREF_3)) |
| 141 | [TMX-GPR30] + [GRK5/6] -> [TMX-GPR30-p2] + [GRK5/6] | k141= 0.59 |  | ([Heitzler et al., 2012](#_ENREF_3)) |
| 142 | [TMX-GPR30-p2] -> [TMX-GPR30] | k142= 0.067 |  | ([Heitzler et al., 2012](#_ENREF_3)) |
| 143 | [TMX-GPR30] + barr2 -> [TMX-barr2-GPR30] | k143= 0.205 |  | ([Heitzler et al., 2012](#_ENREF_3)) |
| 144 | [TMX-barr2-GPR30] -> [TMX-GPR30] + barr2 | k144= 1.05 |  | ([Heitzler et al., 2012](#_ENREF_3)) |
| 145 | [TMX-GPR30-p2] + barr2 -> [TMX-barr2-GPR30-p2) | k145= 1.04 |  | ([Heitzler et al., 2012](#_ENREF_3)) |
| 146 | [TMX-barr2-GPR30-p2] -> [TMX-GPR30-p2] + barr2 | k146= 0.347 |  | ([Heitzler et al., 2012](#_ENREF_3)) |
| 147 | [TMX-barr2-GPR30] + ERK -> ppERK + [TMX-barr2-GPR30] | k147= 4.2E-4 |  | ([Heitzler et al., 2012](#_ENREF_3)) |
| 148 | [TMX-barr2-GPR30-p2] + ERK -> ppERK + [TMX-barr2-GPR30-p2] | k148= 14.44 |  | ([Heitzler et al., 2012](#_ENREF_3)) |
| 149 | [Ga-GTP] + PIP2 -> DAG + [Ga-GTP] | k149= 4.63 |  | ([Heitzler et al., 2012](#_ENREF_3)) |
| 150 | DAG + PKc -> DAG + [PKc-active] | k150= 0.079 |  | ([Heitzler et al., 2012](#_ENREF_3)) |
| 151 | [PKc-active] -> PKc | k151= 1.77 |  | ([Heitzler et al., 2012](#_ENREF_3)) |
| 152 | DAG -> PA | k152= 0.15 |  | ([Heitzler et al., 2012](#_ENREF_3)) |
| 153 | [PKc-active] + SOS -> [Grb2-SOS] + [PKc-active] | k153= 4 |  | KEGG pathway ., estimation |
| 154 | [PKc-active] + [Ras-GAP] -> [Ras-GTP-Ras-GAP] + [PKc-active] | k154= 4 |  | KEGG pathway ., estimation |
| 155 | [PKc-active] + Raf1 -> Raf1active + [PKc-active] | k155= 4 |  | KEGG pathway ., estimation |

**2- rules**

| rule1 | pAkt_total = pAkt + pAktm | Repeated Assignment |
| --- | --- | --- |

**3- Nonzero species:**

| Name | Concentration  (µM) | References |
| --- | --- | --- |
| EGF | 0.008197 | - |
| EGFR | 0.3 | ([Sasagawa et al., 2005](#_ENREF_9)) |
| cbl | 0.8 | ([Sasagawa et al., 2005](#_ENREF_9)) |
| Shc | 1 | ([Sasagawa et al., 2005](#_ENREF_9)) |
| SHP2 | 0.1 | ([Sasagawa et al., 2005](#_ENREF_9)) |
| Grb2 | 1 | ([Sasagawa et al., 2005](#_ENREF_9)) |
| SOS | 0.3 | ([Sasagawa et al., 2005](#_ENREF_9)) |
| Ras-GDP | 0.15 | ([Sasagawa et al., 2005](#_ENREF_9)) |
| Ras-GAP | 0.1 | ([Sasagawa et al., 2005](#_ENREF_9)) |
| Raf1 | 0.5 | ([Sasagawa et al., 2005](#_ENREF_9)) |
| MEK | 0.68 | ([Sasagawa et al., 2005](#_ENREF_9)) |
| ERK | 0.4 | ([Sasagawa et al., 2005](#_ENREF_9)) |
| Pase | 0.5 | ([Sasagawa et al., 2005](#_ENREF_9)) |
| Pase2 | 0.02 | ([Sasagawa et al., 2005](#_ENREF_9)) |
| Pase3 | 0.002 | ([Sasagawa et al., 2005](#_ENREF_9)) |
| ProEGFR | 1 | ([Sasagawa et al., 2005](#_ENREF_9)) |
| EPn | 0.5 | ([Sasagawa et al., 2005](#_ENREF_9)) |
| PI3K | 0.2 | ([Kiyatkin et al., 2006](#_ENREF_7)) |
| TP4 | 0.2 | ([Kiyatkin et al., 2006](#_ENREF_7)) |
| PIP2 | 0.5 | ([Kiyatkin et al., 2006](#_ENREF_7)) |
| Akt | 0.1 | ([Kiyatkin et al., 2006](#_ENREF_7)) |
| PDK1 | 0.1 | ([Kiyatkin et al., 2006](#_ENREF_7)) |
| Takt | 0.1 | ([Kiyatkin et al., 2006](#_ENREF_7)) |
| STAT3c | 1 | ([Yamada et al., 2003](#_ENREF_12)) |
| PP1 | 0.5 | ([Yamada et al., 2003](#_ENREF_12)) |
| PP2 | 0.6 | ([Yamada et al., 2003](#_ENREF_12)) |
| TMX | 250 | MTT result (LC50) |
| GPR30 | 1.0192 | Estimation |
| Gq-trimer | 56.99 | ([Heitzler et al., 2012](#_ENREF_3)) |
| GRK2/3 | 0.899 | ([Heitzler et al., 2012](#_ENREF_3)) |
| Barr1 | 0.858 | ([Ahn et al., 2004a](#_ENREF_1); [Ahn et al., 2004b](#_ENREF_2)) |
| Barr2 | 0.483 | ([Ahn et al., 2004a](#_ENREF_1); [Ahn et al., 2004b](#_ENREF_2)) |
| GRK5/6 | 1.518 | ([Heitzler et al., 2012](#_ENREF_3)) |
| DAG | 0.0090 | ([Heitzler et al., 2012](#_ENREF_3)) |
| PKc | 8.842 | ([Heitzler et al., 2012](#_ENREF_3)) |
| PKc-active | 0.0020 | ([Heitzler et al., 2012](#_ENREF_3)) |

**References:**

Ahn, S., Shenoy, S.K., Wei, H., and Lefkowitz, R.J. (2004a). Differential kinetic and spatial patterns of beta-arrestin and G protein-mediated ERK activation by the angiotensin II receptor. *J Biol Chem* 279(34)**,** 35518-35525. doi: 10.1074/jbc.M405878200.

Ahn, S., Wei, H., Garrison, T.R., and Lefkowitz, R.J. (2004b). Reciprocal regulation of angiotensin receptor-activated extracellular signal-regulated kinases by beta-arrestins 1 and 2. *J Biol Chem* 279(9)**,** 7807-7811. doi: 10.1074/jbc.C300443200.

Heitzler, D., Durand, G., Gallay, N., Rizk, A., Ahn, S., Kim, J., et al. (2012). Competing G protein-coupled receptor kinases balance G protein and beta-arrestin signaling. *Mol Syst Biol* 8**,** 590. doi: 10.1038/msb.2012.22.

Hsieh, M.Y., Yang, S., Raymond-Stinz, M.A., Edwards, J.S., and Wilson, B.S. (2010). Spatio-temporal modeling of signaling protein recruitment to EGFR. *BMC Syst Biol* 4**,** 57. doi: 1752-0509-4-57. doi: 10.1186/1752-0509-4-57.

Huang, C.Y., and Ferrell, J.E., Jr. (1996). Ultrasensitivity in the mitogen-activated protein kinase cascade. *Proc Natl Acad Sci U S A* 93(19)**,** 10078-10083.

Kholodenko, B.N., Demin, O.V., Moehren, G., and Hoek, J.B. (1999). Quantification of short term signaling by the epidermal growth factor receptor. *J Biol Chem* 274(42)**,** 30169-30181.

Kiyatkin, A., Aksamitiene, E., Markevich, N.I., Borisov, N.M., Hoek, J.B., and Kholodenko, B.N. (2006). Scaffolding protein Grb2-associated binder 1 sustains epidermal growth factor-induced mitogenic and survival signaling by multiple positive feedback loops. *J Biol Chem* 281(29)**,** 19925-19938. doi: M600482200. doi: 10.1074/jbc.M600482200.

Rich, R.L., Hoth, L.R., Geoghegan, K.F., Brown, T.A., LeMotte, P.K., Simons, S.P., et al. (2002). Kinetic analysis of estrogen receptor/ligand interactions. *Proc Natl Acad Sci U S A* 99(13)**,** 8562-8567. doi: 10.1073/pnas.142288199.

Sasagawa, S., Ozaki, Y., Fujita, K., and Kuroda, S. (2005). Prediction and validation of the distinct dynamics of transient and sustained ERK activation. *Nat Cell Biol* 7(4)**,** 365-373. doi: 10.1038/ncb1233.

Schoeberl, B., Eichler-Jonsson, C., Gilles, E.D., and Muller, G. (2002). Computational modeling of the dynamics of the MAP kinase cascade activated by surface and internalized EGF receptors. *Nat Biotechnol* 20(4)**,** 370-375. doi: 10.1038/nbt0402-370.

Ung, C.Y., Li, H., Ma, X.H., Jia, J., Li, B.W., Low, B.C., et al. (2008). Simulation of the regulation of EGFR endocytosis and EGFR-ERK signaling by endophilin-mediated RhoA-EGFR crosstalk. *FEBS Lett* 582(15)**,** 2283-2290. doi: S0014-5793(08)00435-3. doi: 10.1016/j.febslet.2008.05.026.

Yamada, S., Shiono, S., Joo, A., and Yoshimura, A. (2003). Control mechanism of JAK/STAT signal transduction pathway. *FEBS Lett* 534(1-3)**,** 190-196.

Yamada, S., Taketomi, T., and Yoshimura, A. (2004). Model analysis of difference between EGF pathway and FGF pathway. *Biochem Biophys Res Commun* 314(4)**,** 1113-1120.
